# AI-Enabled Smart Wristband Providing Real-Time Vital Signs and Stress Monitoring

**DOI:** 10.3390/s23052821

**Published:** 2023-03-04

**Authors:** Nikos Mitro, Katerina Argyri, Lampros Pavlopoulos, Dimitrios Kosyvas, Lazaros Karagiannidis, Margarita Kostovasili, Fay Misichroni, Eleftherios Ouzounoglou, Angelos Amditis

**Affiliations:** 1 School of Electrical & Computer Engineering, National Technical University of Athens (NTUA), 10682 Athens, Greece; 2 Institute of Communication and Computer Systems (ICCS), 10682 Athens, Greece

**Keywords:** smart wristband, wearable, biometric sensor, blood pulse, machine learning, stress detection

## Abstract

This work introduces the design, architecture, implementation, and testing of a low-cost and machine-learning-enabled device to be worn on the wrist. The suggested wearable device has been developed for use during emergency incidents of large passenger ship evacuations, and enables the real-time monitoring of the passengers’ physiological state, and stress detection. Based on a properly preprocessed PPG signal, the device provides essential biometric data (pulse rate and oxygen saturation level) and an efficient unimodal machine learning pipeline. The stress detecting machine learning pipeline is based on ultra-short-term pulse rate variability, and has been successfully integrated into the microcontroller of the developed embedded device. As a result, the presented smart wristband is able to provide real-time stress detection. The stress detection system has been trained with the use of the publicly available WESAD dataset, and its performance has been tested through a two-stage process. Initially, evaluation of the lightweight machine learning pipeline on a previously unseen subset of the WESAD dataset was performed, reaching an accuracy score equal to 91%. Subsequently, external validation was conducted, through a dedicated laboratory study of 15 volunteers subjected to well-acknowledged cognitive stressors while wearing the smart wristband, which yielded an accuracy score equal to 76%.

## 1. Introduction

The monitoring of state of health in humans is a field of significant scientific and research interest, especially as awareness regarding wellbeing has increased. Wearable devices are widely used as a convenient means of providing monitoring of vital signs, such as heart rate and physiological activities. Specific physiological conditions entail increased computational complexity, due to their nature, and require artificial intelligence to be efficiently assessed. One of these conditions is psycho-physical stress.

Stress is defined as a person’s physical, mental, and emotional reaction to certain stimuli that cause strain. Such stimuli are often known as “stressors” [1], and are basically a biological response of the human body to any situation that requires attention or action. Depending on the severity and the timing of the stressor, the produced stress can seriously affect the human organism physically and emotionally, by causing cardiovascular diseases, such as high blood pressure and tachycardia, psychological disorders, and even life-threatening results [2]. Stressors are categorized based on the reactions they cause, and are divided into two fundamental categories: (a) physiological stressors; and (b) psychological/mental stressors. Life-threatening events related to extreme weather phenomena, catastrophes, and various emergencies are both physiological and emotional stressors of high severity, capable of stressing anyone: thus, they are often referred to as “absolute stressors” [3]. An example of such an event is the evacuation of a ship during an emergency situation, which is the research interest of the SafePASS project, and the main reason for implementing the work presented in this paper.

The evacuation of a large passenger ship is a safety-critical and strictly time-bound task, which typically involves thousands of people moving within parts of the ship. The process involves complex and critical decision making, based on the situation on board, and the information available to the decision makers (i.e., navy officers and cabin crew) [4]. To tackle the above challenges, and to assist in the optimization of a ship’s evacuation process, the EU-funded SafePASS project has introduced easy-to-use and low-maintenance Personal Survival Equipment (PSE), such as smart lifejackets and smart wristbands, which are capable of operating in emergency conditions, and which interact with one another. This work presents the design and development of a fully-customized smart wristband, and the integration of a machine learning model to detect stress incidents. The latter is obtained through ultra-short pulse rate variability executed on the microcontroller of the device.

### 1.1. Related Work

The development of smart wearable devices for monitoring human physiology and assisting in the betterment of people’s lives has been the subject of several research works over the years. An indicative approach, aimed at providing real-time sweat alcohol monitoring, was recently presented in [5]. To design the low-cost and non-invasive breathalyzer in the form of a smart wristband, a microcontroller, equipped with BLE and Wi-Fi interfaces, and a sweat alcohol metal oxide (MOX) were integrated together, and achieved accuracy of 94.66% in detecting sweat alcohol concentrations. The COVID-19 pandemic triggered several related works, such as the one by Mahapatra et al. [6], who developed a GPS-enabled wristband as a part of an automatic tracking and contact tracing system for people, and Khairam et al. [7], who proposed a sensing bracelet with an ultrasonic sensor and thermometer, to determine physical distancing, and to assist the containment of the infection.

Regarding stress detection, while several works—such as [8,9,10,11]—have been based on the processing of signal segments shorter than 5 min, the potential of stress detection based on signal segments shorter than 1 min has been rather under-researched. In this section, the indicative literature covering both unimodal and multi-modal approaches has been included despite the fact that the latter—i.e., exploiting multiple signals for stress inference—would probably come at the cost of higher computational complexity induced on the microcontroller of the developed embedded device. However, focus has been placed on ultra-short processing—i.e., processing based on signal segments lasting less than 5 min—which has been deemed the most viable solution for meeting the existing requirements for the immediate display of ML-enabled inference conducted on the layer of the microcontroller. Within that scope, selected results found in pertinent state-of-the-art research are reported below.

In the field of wearable-based stress detection, it is often the case that off-the-shelf, commercial solutions are utilized to acquire biometric data, and that the collected datasets are subsequently used for tackling the stress detection task. For instance, Nath et al. [12] used four physiological signals—electrodermal activity (EDA), premature ventricular beats (PVB), interbeat intervals (IBI), and skin temperature (ST)—provided by a wrist-worn device, to develop a stress detection model for the elderly. During their experiment, salivary cortisol was used as a clinical biomarker for measuring the stress of 40 participants. The proposed model achieved an accuracy of 94% in distinguishing stress, and no stress states when all four signals were used. Under the same scope, Vila et al. [13] tried to predict the stress levels experienced by travelers over a long journey, by proposing a personalized regressive model based on several biosignals. Specifically, accelerometer measurements, EDA, blood volume pulse (BVP), and ST signals, recorded by the same wristband device, were used as inputs. The proposed solution managed to correctly detect 92.6 to 100% of all the reported stress-less time windows, depending on the participant’s level of activity. In both of these cases, signal processing, feature extraction, and machine learning computations were executed in a secondary and external device, and were not in the wearable device itself, as has been achieved in the presented work.

There is also relevant research putting special emphasis on the length of the signal segment used for stress inference. In a recent study, Lee et al. [14] conducted heart rate variability (HRV) analysis, using various time lengths, and reported a minimum signal length of 2 min to detect stress via frequency domain analysis. An accuracy equal to 90.5% was reported in the context of leave-one-subject-out cross-validation. In another study, Castaldo et al. [9] explored the validity of ultra-short HRV features as surrogates of short HRV features, to detect mental stress in a real-life scenario using machine learning methods. Specifically, a subset of HRV-extracted features was selected to display consistency across all of the signal segment lengths (from 5 min segments to 1 min segments), and achieved accuracy of 88% in a subject-independent context.

A few selected works, of special interest and relevance to the research presented here, are reported below. Firstly, Schmidt et al. [15], who introduced the Wearable Stress and Affect Detection (WESAD) dataset. In the binary case (stress vs. non-stress), accuracy scores of up to 93% were reached with 1-minute-length signal segments, which were obtained by using all chest modalities (i.e., ACC, ECG, EDA, EMG, RESP, and TEMP). The unimodal PPG-based version of the stress model obtained a nearly 86% accuracy score using an LDA classifier. It was concluded that data generated from a chest-based device lead to higher model performance compared to data generated from wrist-based devices. Secondly, the approach of Salai et al. [16] has been considered of high interest, as presenting a method opting for low computational complexity, and detecting stress using three ECG-extracted time domain features while obtaining satisfactory accuracy of 74.6%. Having developed this lightweight approach, the authors in [16] claimed that it could be efficiently implemented on mobile devices—without, however, having proceeded with any integration whatsoever. Golgouneh and Tarvirdizadeh [17] took an interesting stress detecting approach, which shared several aspects with the work presented here. Specifically, the authors in [17] fabricated a portable device, made use of ultra-short signal segments for detecting stress levels, and tested their approach on an independent dataset consisting of 16 volunteers. However, the device they used limited the subject’s mobility, and collected both PPG and GSR signals, constituting a multi-modal approach; also, the provided algorithm was not executed on an embedded system. A classification accuracy of 75% was obtained, using the implemented KNN classifier. This outcome was very satisfactory, given that it came up through an independent validation process, and that the algorithm performed three-label classification.

For a general overview of the field, the interested reader is referred to [18,19,20], and to references therein: these constitute comprehensive and well-researched review articles, investigating stress detection approaches based on wearable sensors in the context of different tasks, such as driving, studying, working, etc. In [18], meaningful guidelines, with respect to crucial aspects of stress detection studies—such as stress elicitation, ground truth acquisition, etc.—were also identified. Overall, it is observed that researchers often tend to report performance obtained within the context of cross-validation, and not based on a separate held-out dataset. Of course, this is not always the case, with several works testing their classifiers in a completely separate dataset, e.g., in [9].

### 1.2. Contributions

To the best of our knowledge, this is the first work to introduce a lightweight stress-detecting solution that is already embedded into the microcontroller of a wearable device, and operates in real time. Instead, pertinent approaches have been strictly limited to the modeling part of the stress detection task, with no reference to the potential integration of the solution, have integrated their solution into a portable but not wearable device [17,21], or claim that their approach is lightweight enough to be integrated, in the future, into a wearable device [10,16]. Additionally, most studies have focused on developing a method for providing reliable stress detection based on short-term heart rate variability or pulse rate variability features, extracted from portable devices [22,23,24,25]. A few have attempted to detect stress based on ultra-short signal segments, but mostly not shorter than 1-min long [9,26,27]. The present work contributes to the under-researched ultra-short PRV-based method of stress detection, by exploiting 30-s PPG segments to generate real-time on-device inference. At this point, it should be noted that a meaningful comparison among different works in wearable-based stress detection is far from straightforward, due to lack of standardization regarding data collection protocols (stressors applied, ground truth acquisition, etc.), and also due to the fact that several works report performance achieved in a cross-validation context alone. The presented approach reports the performance with respect to an independent laboratory study using a similar, yet far from identical, data collection protocol and a different device (i.e., a fully custom-made device developed by the authors). The study included 15 participants, and was conducted for external validation purposes.

Taking all the above into consideration, the presented work provides the following contributions:

- the development of a low-cost, easy-to-use, and fully customized smart wristband, designed properly for emergency events like the evacuation of a ship;
- a real-time ultra-short pulse rate variability process, conducted on the smart wristband, based on 30 s PPG signal segments;
- the implementation of a lightweight machine learning pipeline for stress detection, using an algorithm based on five time domain features and one extra heart-rate-related feature, to provide a “stress” or “no stress” output;
- real-time stress detection by integration of the ML pipeline into the embedded device;
- a two-stage evaluation of the proposed system: firstly, a 91% accuracy score was obtained on a previously unseen subset, held out from the cross-validation process; secondly, a 76% accuracy score was achieved in the context of an external validation process performed through a dedicated laboratory study.

## 2. Materials and Methods

### 2.1. Biosignals

Biosignals are time-dependent measures of biological processes occurring in the human body, and can be utilized to infer a person’s state of health. Biosignals have been shown to be efficient as indicators of stress [28,29]. Their reliability is based on the fact that they are not subject to intentional or even partial conscious control, unlike the more manipulable behavioral and psychological components of stress. Various detectable biosignals of the human body have been utilized throughout the years: the ones that have been proven to be more reliable and widely used to extract health state indicators, such as stress, are described below:

- Electrocardiogram (ECG): an ECG measures the electrical activity generated by the heart as it contracts. The ECG is one of the most extensively used signals in stress detection research [30,31], because it directly reflects the activity of the heart, which in turn is affected by Autonomic Nervous System (ANS) changes. The characteristic peaks of the ECG are denoted by the letters P, Q, R, S, and T. The R-peak (i.e., the most distinctive peak) is considered crucial, and most analyses exploit the distribution of the time elapsed between two successive R-peaks—usually called RR intervals (RRI) or interbeat intervals (IBI) [32].
- Photoplethysmography (PPG): PPG is a simple optical technique used to detect volumetric changes in blood in peripheral circulation [33]. PPG is a low-cost and non-invasive method that makes measurements at the surface of the skin [34]. From PPG signals, various measures can be extracted, such as pulse rate (PR), pulse rate variability (PRV), blood volume pulse (BVP), blood oxygen saturation level ($SpO_{2}$), and blood pressure (BP). BVP is the signal produced as a result, when filtering the PPG signal with a band-pass filter. The selection of the corresponding cut-off frequencies is rather arbitrary: a low cut-off frequency of 0.5 Hz and a high cut-off frequency between 3–5 Hz are typically used, considering both the lowest HR at rest (30 bpm) and highest HR (180–300 bpm) [35,36].
- Electromyogram (EMG): an EMG signal is a biomedical signal that measures electrical currents generated in muscles during their contraction. Stress impacts muscle contraction, which is why EMG can be exploited to identify stress [37].
- Electrodermal Activity (EDA): EDA, also called Galvanic Skin Response (GSR) or skin conductance (SC) is a measure of changes in the electrical conductance of skin, based on the production of sweat. It is widely used in psychological stress detection [38,39].

Another well-acknowledged indicator of stress, which is not a biosignal but a biosignal-extracted measure, is heart rate variability [40]. Heart rate variability (HRV) reflects the distribution of heartbeats over a given time interval. HRV can be obtained by computing the time difference corresponding to each pair of successive peaks, and it represents one of the most promising markers of the ANS [41]. HRV-extracted metrics are commonly used as features in stress detection tasks, as they are considered reliable indicators of stress [42,43]. Ideally, HRV analysis would require the acquisition of the ECG signal; however, PPG-derived HRV analysis—often called Pulse Rate Variability (PRV) analysis—is considered to be an interesting alternative, as PPG is a more convenient and less intrusive measurement technique. In fact, PPG has been used for HRV parameters estimation [44], and it seems to obtain high temporal peak agreement with ECG-based HRV analysis. However, although HRV and PRV were found to be highly correlated, they could not be considered identical [45].

As described in Section 1, the primary purpose of this endeavor was to design a wearable device enabling real-time biometrics monitoring and stress detection in the context of a very challenging use case, such as the evacuation of a passenger vessel in an emergency. It is evident that such scenarios entail serious usability constraints, regarding the safety and free movement of the wearer. Signals such as ECG, EEG, and EMG were excluded as impractical, because in most cases, cables and electrodes across the body were required.

Based on the aforementioned conclusions, we decided to adopt a unimodal approach, and to proceed with the implementation of a stress detection model exclusively based on a signal reflecting cardiac activity. We focused on investigating sensors for acquiring a PPG signal, as this was a non-obtrusive method that could be used for the extraction of important biometrics and indicators, such as PR, $SpO_{2}$, and PRV.

### 2.2. System Design and Architecture

The assessment of the system requirements was an essential step in the design process of the smart wristband, in order to proceed with its development. The system requirements defined by the SafePASS project are as follows:

1. real-time monitoring of stress, and specific biometric measurements (heart rate and oxygen saturation) of the passenger during an emergency evacuation event;
2. transmission of the acquired measurements to a nearby device (smart lifejacket, smartphone) via a Bluetooth Low Energy (BLE) communication protocol;
3. unique identifier and pairing option using Near-Field Communication (NFC) protocol;
4. operational life of at least 3–4 h continuously;
5. a non-obtrusive and safe device;
6. a miniaturized wearable and user-friendly device.

**Figure 1:**
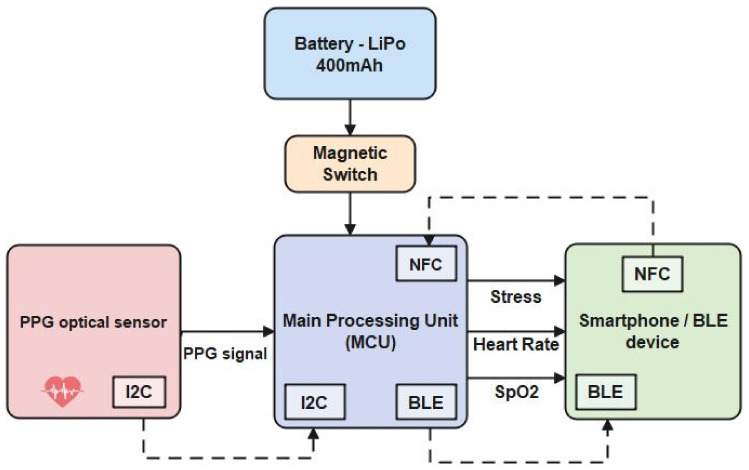
System architecture.

After analyzing all system requirements, the system architecture was structured. The architecture is presented in Figure 1, and its components are described in the following Table 1:

#### 2.2.1. Hardware Components

The component selection for the smart wristband was based on the following criteria: (a) functionalities; (b) reliability; (c) compatibility; (d) availability; and (e) cost.

**Figure 2:**
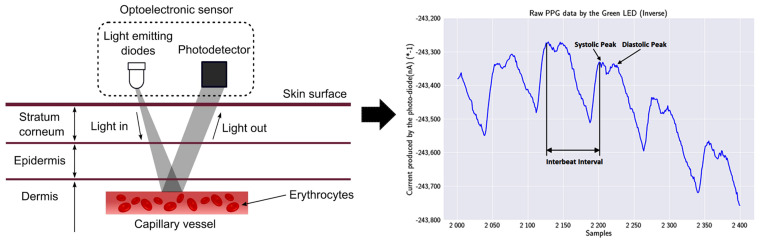
PPG technique and output signal: on the left, a LED of the PPG sensor emits light into the wrist’s skin, and the photodetector absorbs the reflection; on the right, the output signal of the used MAX30101 sensor, inverted to reflect the correct morphological representation, with visible systolic and diastolic points [48].

The finding of a reliable PPG technology-based sensor was a priority for acquiring the heart’s biosignal, since it was concluded to be the most appropriate technology. A PPG technology-based sensor utilizes light-emitting diodes (LEDs) to cast light on a person’s skin (i.e., wrist). As shown in Figure 2, the emitting light travels through the biological tissues, and is absorbed by the skin, the bones, and the veins/arterial blood. The reflected light that returns is acquired by a photo-detector: thus, the amplitude of the PPG measurement is proportional to the received light intensity.

Keeping all the above in mind, the MAX30101 sensor by Analog Devices [46], which provides a single packaged module including all the required optical elements, was selected. The required elements were three integrated LEDs (red LED at 670 nm wavelength, IR LED at 900 nm wavelength, and green LED at 545 nm wavelength) and two photodetectors, plus noise filters with ambient light rejection, and analog-to-digital converters for I2C communication with the MCU.

For the main processing unit, an nRF52840 SoC microcontroller by Nordic Semiconductors, Trondheim, Norway [47], based on a powerful ARM Cortex-M4 CPU, was selected. The specific MCU integrated both BLE and NFC communication interfaces, and had enough computational and memory resources to host the ML model.

#### 2.2.2. PCB and Enclosure Design

In order to achieve the development of a device that was composed of all the hardware components described above, while also being wearable, safe, and reliable, we proceeded with the design of a PCB. The requirement for placement stability of the sensor on the wrist also entailed the need for a PCB board, as it has been observed that the reliability and quality of the acquired data from a PPG sensor can be significantly affected by movement, especially on the wrist [49].

The designed PCB was double-sided, with the top side hosting all the components described in Section 2.2.1, except the PPG sensor, which needed to be in touch with the passenger’s wrist, and was therefore placed on the bottom side. With the implementation of the PCB board, we managed to meet a number of system requirements, and to provide:

- miniaturization: due to the fact that the wristband is placed on a person’s wrist during an emergency event, and needs to be convenient for them, the limitations of size, weight, and available space for placement were evident; respecting those limitations, the smallest possible components were chosen, and the electrical routing of the board was made in such a way as to reduce the overall size;
- reduced cost: the use of the standalone SoCs, and not their development kits, reduced significantly the overall cost, because the production cost was paid once, and not for each development kit; furthermore, relatively low-cost but still reliable components were chosen for that reason;
- increased reliability: the electrical connections and routing of the components on a PCB were proven to be more reliable than those made by human hands and solder masks;
- mass production: after completing the PCB design, the mass production of the boards could be outsourced and completed faster, producing identical boards.

**Figure 3:**
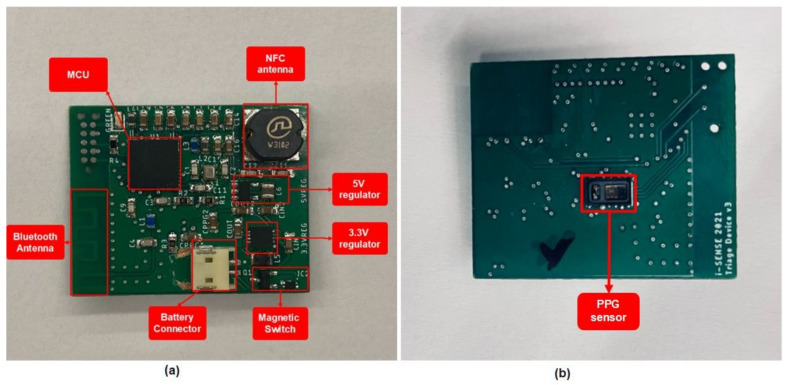
PCB after the SMT assembly, with all the necessary components: (**a**) top side; (**b**) bottom side.

The construction of a total of 15 PCB boards was completed by placing the SMT components accurately, and using a reflow oven to solder them. In Figure 3, an assembled board is presented. These boards were used not only in the validation of the stress detection model, but also in the pilot demonstration of the SafePASS EU project, as a preliminary test.

**Figure 4:**
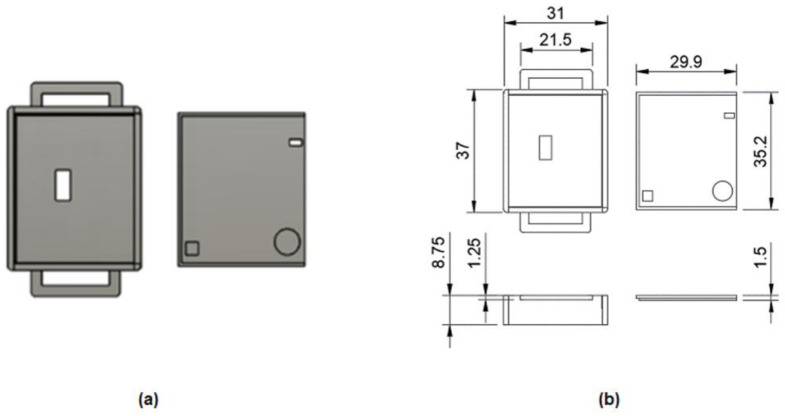
3D design of the enclosure (casing and sliding lid): (**a**) mechanical 3D design; (**b**) mechanical drawing with dimensions.

**Figure 5:**
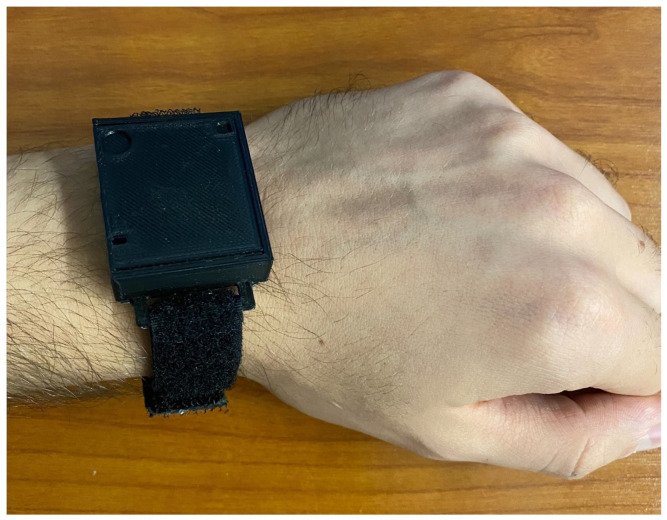
Smart wristband on the wrist.

The enclosure of the developed board was custom-designed and 3D printed using thermoplastic polyurethane (TPU) as 3D filament. The specific material was chosen for its flexibility and sufficient compactness, which made it suitable for wearable devices. The mechanical drawing of the enclosure is presented in Figure 4. For the straps that were used to tie the device to the wrist, adhesive velcro tapes were used, for convenience. As depicted in Figure 5, the final outcome was a small and lightweight (18 g) device that was wearable on the wrist.

The relatively low cost was another milestone during the development of the smart wristband. We managed to complete this milestone at an estimated cost of around 40 euros per unit, by selecting low-cost components and the custom design of the PCB and enclosure.

#### 2.2.3. Software

As a consequence of the system requirements, the firmware of the smart wristband needed to incorporate multiple functionalities. The main functionalities were:

- tuning and data allocation from the biometric sensor;
- calculation of the pulse rate from the raw PPG data;
- calculation of the $SpO_{2}$ from the raw PPG data;
- processing of the PPG signal, including low-pass filtering;
- execution of the ML stress detection model;
- formatting data following the standard BLE profiles for transmission;
- NFC type A tag support;
- power consumption optimization.

**Figure 6:**
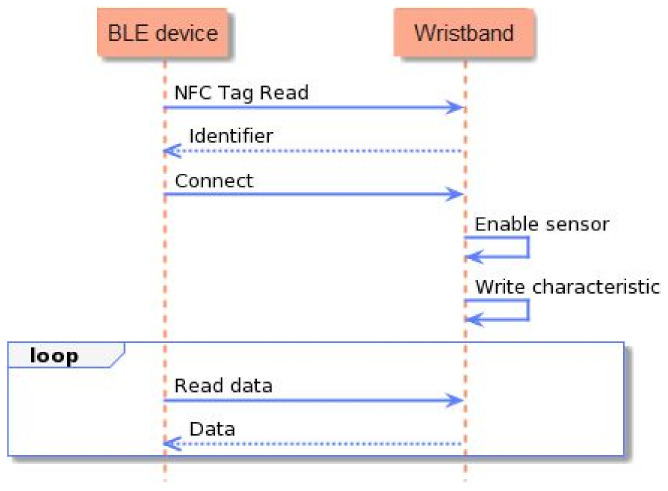
Process flow of the smart wristband.

To implement all the aforementioned functionalities, the development of the smart wristband’s firmware involved task scheduling, memory and power management, and appropriate data formatting. To achieve stability, and to surpass the challenges, the firmware was developed over the Zephyr open-source real-time operating system (RTOS) [50], which was lightweight and compatible with the selected MCU architecture. The process flow and the interaction of the smart wristband with a BLE gateway device are outlined in Figure 6. In detail, after the initialization of the MCU and the global parameters were completed, the NFC tag functionality was established, and the CPU went into low-power mode, until a device with an NFC reader came close to the smart wristband. Once the NFC communication at 13.56 MHz frequency was initiated, the smart wristband transmitted its MAC address to the BLE client device (i.e., the smart lifejacket), and enabled the BLE interface to complete the pairing. Simultaneously, communication with the biometric sensor was established, and the acquirement of the measurements was initiated. These PPG measurements were gathered and stored on a buffer, for a duration of 30 s. At a sample rate of 50 Hz, this resulted in a buffer with a size of 1500 measurements, which represented the function of the heart. This was followed by the processing and filtering of the signal, and the calculation of the pulse rate and oxygen saturation level ($SpO_{2}$). The signal was also imported to the ML stress detection model, based on which, the inference was conducted, and a binary value—0 for a non-stressful event, and 1 for a stressful event—was returned. Once the inference was completed, the data were formatted, based on the standard Bluetooth profiles [51] (Pulse Oximeter Profile 1.0.1 for $SpO_{2}$, Heart Rate Profile 1.0 for heart rate, and custom-made profile for stress detection output), and transmitted via BLE. Following the successful transmission, the buffered data were freed, and a new cycle of 30 s of measurements acquirement was initiated. The calculations of the pulse rate and the oxygen saturation are described in Section 3, and the stress detection model is presented in Section 4.

## 3. Biometrics Detection Methodology

The tuning and configuration of the biometric sensor was a process that proved to be of significant value, with respect to the obtained measurements and, as a consequence, to the results. Specifically, it was observed that the signals were not always sufficiently stable, and that the systolic point, which is normally the first and highest peak of the PPG signal, coincided with the diastolic peak. This fact reduced the quality of the signal, and produced some wrong interbeat interval calculations, which led to reduced heart rate and $SpO_{2}$ accuracy. This phenomenon basically appeared during the movements of the wrist. In order to reduce this artifact, we focused on experimenting with the two basic parameters that appeared to mostly affect the signal:

- sampling rate: the equipped sensor provided a configurable sampling rates of 50, 100, and 200 Hz. An increased sample rate led to a clearer and more accurate signal, as the potential erroneous measurements had a lower impact. For that reason, we increased the sensor’s sample rate to 200 Hz, and utilized its sample averaging capability every four samples, to reduce the overall signal size. As a result, we achieved a final sampling frequency of 200/4 = 50 Hz.
- pulse amplitude: increased pulse amplitude led to a more stable PPG signal, due to the fact that an emitting LED, with higher light intensity, evoked increased light absorption by the wrist, and made the produced PPG signal less prone to movements. On the other hand, the increased pulse amplitude required more power consumption; thus, only the green LED’s pulse amplitude was increased and configured at 40.032 mA, while the red and IR LEDs were set at 20.6 mA.

The best results were produced using the configurations presented in Table 2 for each LED.

Although the green LED produced a lower amplitude signal, it demonstrated increased stability in regard to the artifacts caused by wrist movements and, for that reason, it was deemed the most suitable option for the calculation of the heart rate. The $SpO_{2}$’s calculation required red and infrared LEDs, due to the fact that the absorption of light at these wavelengths differs significantly between blood loaded with oxygen and blood lacking oxygen.

### 3.1. Pulse Rate Algorithm

**Figure 7:**
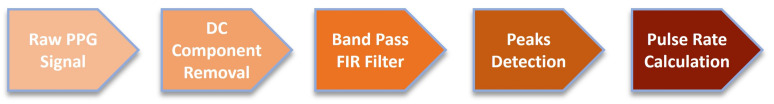
Pulse rate calculation pipeline.

For the accurate calculation of the pulse rate from the PPG signal, specific signal processing steps, outlined in Figure 7, were implemented on the acquired raw data produced by the sensor’s green LED. Firstly, the raw data incorporated a DC component, which had to be removed. The signal processing method used to remove the DC component was the initial calculation of a moving average, and then the subtraction of it from the delayed version of the input signal. The number of samples used to calculate the moving average depended on the sample rate of the signal. An average of the samples that included between one and two heartbeats proved to be more efficient. As a result, with the sample rate at 50 Hz, and the fluctuation of time between two beats at 0.33–1 s, an average of every 64 samples (the closest power of 2) was computed, which was approximately 1200 ms.

**Figure 8:**
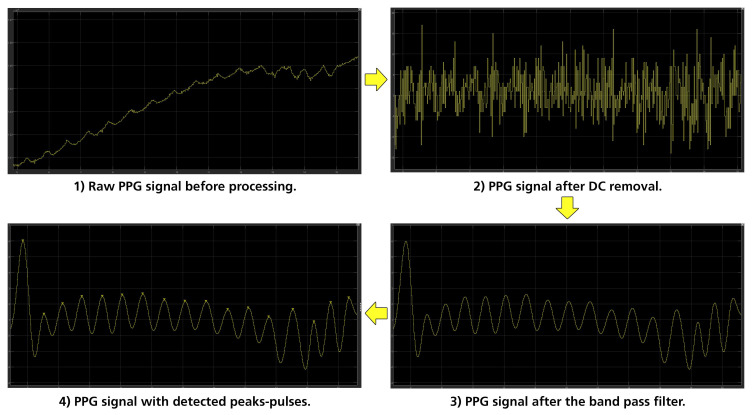
Stages of PPG processing.

The pulse rate of a human being at rest can be as low as 30–40 bpm, and can reach near to 220 bpm in intense exercise or tachycardia, which corresponds to pulse rate frequencies of 0.7 Hz to 3.5 Hz [36]. For these reasons, the noise generated from different frequencies—especially the higher ones, which are more evident—had to be removed. The signal processing method selected to achieve this was a finite impulse response (FIR) filter and, specifically, a band pass one with lower and upper cut-off frequencies of 0.7 Hz and 3.5 Hz, respectively. To properly design the FIR filter, the Parks–McClellan algorithm was implemented for the calculation of the filter’s coefficients. The Parks–McClellan algorithm uses the Remez exchange algorithm and Chebyshev approximation theory to design filters with an optimal fit between the desired and actual frequency responses. The created filters are optimal, in the sense that the maximum error between the desired frequency response and the actual frequency response is minimized [52,53]. An example of the PPG signal processing is shown in Figure 8.

The next step was finding the filtered signal’s peaks, using the local maxima method with real-time implementation. The fact that the human heart rate varies between 30 to 220 beats per minute, results in the time intervals between two heartbeats (signal peaks) being between 2 s and 0.27 s. The configured sampling frequency of the PPG sensor was 50 Hz, which entailed that the number of samples between the two peaks was from 48 to 12, respectively. In the most demanding scenario, with a heart rate of 220 beats per minute, the samples between the local minimum and the next local maximum were half the number of samples, so approximately 6–7 samples. For this reason, the search range segment we decided to use was 7 samples [54]. This meant that every new sample was compared with the last 7 samples. If a change in the monotony of the signal based on the previous samples was observed, and the current sample each time was greater than the previous 7 samples, it was characterized as a local maximum, and saved. Finally, by comparing the saved local maxima between them, it was decided whether or not a local maximum was a systolic peak, and thus a heartbeat. During this process, the device counted the number of heartbeats that had been detected within the 30 s, and then calculated the beats per minute by multiplying them by 2. The selection of the window length was not straightforward, but rather was based on pertinent literature suggesting that physiological features are typically aggregated over 30 to 60 s signal segments [55]. The authors proceeded with the selection of the lowest limit within the acceptable range (30 s), taking into consideration that the longer a signal segment was, the higher the probability of motion artifacts occurring, especially during a ship evacuation in an urgent event.

**Figure 9:**
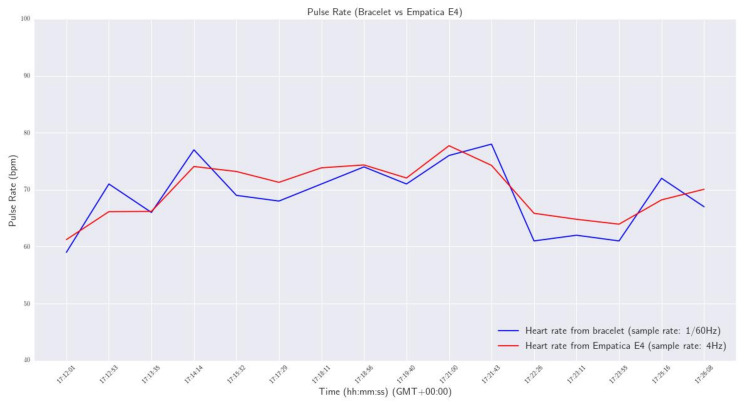
Pulse Rate - Smart wristband vs Empatica E4.

In order to investigate the validity of the calculations across the spectrum of the human heart rate range, a series of tests were conducted. At first, a signal with predefined peaks was generated—using the Matlab Simulink software, https://www.mathworks.com/products/simulink.html, accessed on 13 December 2022—and was imported to the wristband, to check if the device detected correctly all the predefined peaks. After the successful completion of this task, the device’s real-time functionality on a human wrist was investigated. For this reason, a small-scale sanity check was conducted, by comparing the pulse rate provided by our smart wristband with the one measured by a commercial Class 2A medical wristband (Empatica E4, https://www.empatica.com/en-eu/research/e4/, accessed on 13 December 2022). Specifically, a volunteer agreed to wear, at the same time, our device on his left hand, and the commercial one on his right hand, for approximately 15 min in a laboratory environment, to monitor his pulse rate. The sampling rate of the developed wristband was configured at one measurement per 60 s (0,17 Hz), and the E4 at default 4 Hz, so the mean of every 240 values was calculated in the case of E4. The acquired data are presented in the plot of Figure 9.

From the results, it was observed that the maximum deviation of pulse rate measurements between the two devices was 2.3 bpm, and the maximum calculated difference was 4.76 bpm.

### 3.2. $SpO_{2}$ Algorithm

**Figure 10:**
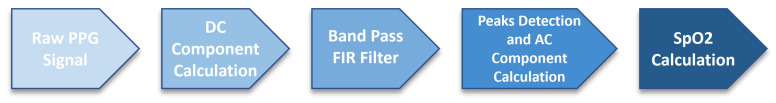
$SpO_{2}$ calculation pipeline.

For the calculation of the oxygen saturation level on the passenger’s wrist, also known as $SpO_{2}$, a similar signal processing pipeline was followed as depicted in Figure 10. In the blood, oxygen is transported by hemoglobin (Hb), which absorbs light in different wavelengths. There are two main forms of Hb in blood: oxygenated hemoglobin (HbO_2_), which carries oxygen, and deoxygenated hemoglobin (RHb). The hemoglobin that carries oxygen causes different levels of light attenuation from the hemoglobin without oxygen. For this reason, two different lights were used: an infrared (IR) LED and a red LED, in whose wavelengths the absorption of the light from the blood differed significantly. According to the bibliography [56,57], $SpO_{2}$ can be measured by the ratio of the changing absorbance between the RED and IR light emitters on the hemoglobin.

**Figure 11:**
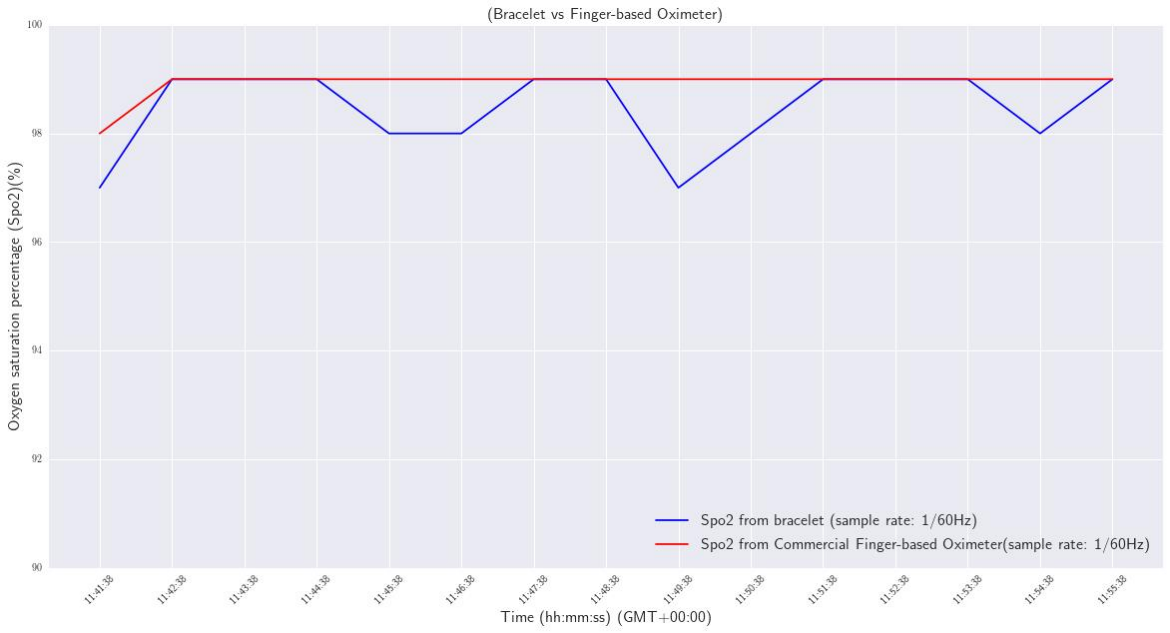
Oxygen saturation percentage: smart wristband vs commercial finger-based oximeter.

The photo-diode of the sensor absorbs the reflected light from both LEDs (red and IR), and generates a proportional current measured in nA, which expresses the energies of the absorbing lights. These generated currents are the raw PPG data, which contain an AC and DC component, and return them to the DSP module for digitization and ambient noise cancellation. From these measurements, the oxygen saturation in the blood can be calculated. In our case, two PPG signals, consisting of one hundred raw values from PPG for Red and IR LEDs, were stored, and then the DC component of every signal was calculated. For the calculation of the DC component, the moving average method was used, as described in the pulse rate calculation, but without removing it this time from the signals. The next step was to pass the two signals from the same FIR band-pass filter, with the lower cut-off frequency at 0.7 Hz and the upper one at 3.5 Hz, to remove the unwanted frequencies. Following this, the peaks in each signal were identified, and the calculation of the AC values was based on the former. To define the $SpO_{2}$, the rate between the AC and DC components of every signal was calculated. The oxygen saturation percentage was the ratio between these two rates, as expressed in Equation (1):

(1) $$SpO_{2}=aR^{2}+bR+c$$

where *R* is defined by the following equation:

(2) $$R=\frac{AC_{red}/DC_{red}}{AC_{ir}/DC_{ir}}$$

and *a*, *b*, and *c* are calibration coefficients defined by the standards of the U.S. Food and Drug Administration (FDA, https://www.fda.gov/, accessed on 17 December 2022). A first evaluation process was completed as a sanity check, using a commercial and clinically validated finger-based oximeter (BRAUM, YK-81CEU, https://www.braunhealthcare.com/za_en/pulse-oximeter-1, accessed on 26 January 2023). Specifically, a volunteer wore the developed smart wristband on his left wrist, and placed his index finger in the commercial oximeter for 15 min, to measure his oxygen saturation level. The sample rate for both devices was one measurement per minute (1/60 Hz). The results are plotted and compared in Figure 11.

From the results, it was observed that the maximum deviation of $SpO_{2}$ measurements between the two devices was 2%, and the maximum calculated difference was 0.4%.

### 3.3. Stress Detection Model

**Figure 12:**
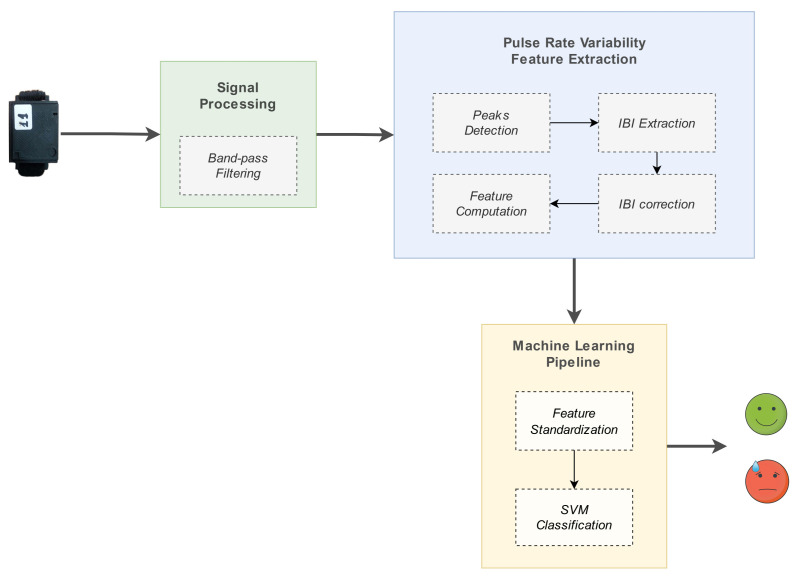
Stress detection model pipeline.

One of the main purposes of the work presented in this paper was the implementation of a model for the detection of stress instances experienced by the person wearing the device (i.e., the passenger on the ship). The development of such a model was a challenging process, due to the complexity of the problem, and also the resource-constraining nature of the case study of interest. The suggested pipeline is outlined in Figure 12, and consisted of a chain of three main processing elements:

1. signal preprocessing: filtering of the raw PPG signal and peaks detection, to enable subsequent IBIs calculation;
2. PRV feature extraction: IBIs calculation and generation of relevant input features;
3. machine learning processing: ML algorithm used for tackling the problem as a binary classification task (’stress’, ’no stress’).

The components involved in the stress detection system are described in detail in the corresponding subsections.

#### 3.3.1. Dataset

The wearable stress and affect detection (WESAD) public dataset [15] was selected for establishing a machine learning pipeline for automatic stress detection, as a very well-established dataset that several works have been based upon. The WESAD dataset was generated based on a well-rounded data collection protocol, and made use of a medical-grade wearable device (Empatica E4) as a ground truth generator. The WESAD dataset is available at the UCI data repository, and provides physiological and motion data, recorded both from medical-grade wrist devices, (https://www.empatica.com/en-eu/research/e4/, accessed on 14 December 2022), and from chest devices (https://www.pluxbiosignals.com/collections/wearables/products/cardioban, accessed on 14 December 2022), worn by 15 subjects during a lab study. Specifically, the following sensor modalities are included: blood volume pulse; electrocardiogram; electrodermal activity; electromyogram; respiration; body temperature; and three-axis acceleration. The decision to proceed with a unimodal approach exploiting the PPG signal was based on the pertinent literature, and the consideration of the distinct, resource-constraining nature of the use case of interest.

#### 3.3.2. Preprocessing

The next step, following data acquisition, was to filter the raw PPG signal. Proper preprocessing of the input signal has been proven to have a significant impact on the subsequent signal analysis and performance of machine learning models [58,59], especially in the case of biosignals [60]. The main purpose of the preprocessing phase is to remove the noise from the acquired signal, which in our case was mainly caused by the artifacts from the wrist movements and the ambient light. To this end, the preprocessing was conducted in three stages: (a) DC component removal; (b) bandpass filtering; (c) systolic peak detection. The first two stages were identical to the ones followed for the pulse rate computation, as described in Section 3.1. The additional and final step was to store the location indices of the detected systolic peaks into a buffer, to be subsequently fed into the feature extraction component.

#### 3.3.3. Feature Extraction

The goal of the feature extraction process was, given the output of the previous step—i.e., an array of PPG systolic peak location indices for a given 30 s PPG signal segment—to generate a minimal, yet efficient, set of features. This goal had to be obtained by keeping computational complexity and processing power at a manageable level while, in parallel, enabling the provision of reliable stress detection. Normally, PRV metrics can be calculated using time domain, frequency domain, and non-linear measurements. Time domain indices of PRV quantify the extent of variability in measurements of the interbeat intervals (IBIs), i.e., the time elapsed between successive heartbeats. Frequency domain measurements estimate the distribution of absolute or relative power into four frequency bands. Finally, non-linear metrics reflect the unpredictability of a time series. For an overview regarding widely-used HRV time domain, frequency domain, and non-linear metrics, the reader is referred to Shaffer and Ginsber [61]. For the needs of the specific use case, time domain measurements were considered the most suitable approach, given that all computations should be performed on the layer of the microcontroller, and also that frequency domain features are greatly affected by the selected window length [62]. Performing PRV analysis on 30 s segments of PPG signal constituted the so-called ultra-short-term HRV analysis—not to be confused with its short-term (>5 min) and long-term (24 h) counterparts, with the latter representing the ’gold standard’ for clinical HRV assessment [61]. Although results from long-term HRV analysis are not identical to those produced by its short-term and ultra-short-term analyses, it is suggested by the pertinent literature that ultra-short-term HRV analysis is a good surrogate method by which to assess patterns in HRV measures [63]. IBIs were obtained as the temporal difference of the systolic peak locations. The ectopic beats effect was alleviated by using standard Z-score cut-off values, and replacing outliers with the median IBI value. The calculated time domain PRV parameters are listed in Table 3.

#### 3.3.4. Machine Learning Pipeline

This section describes all the machine learning investigations (training, hyper-parameter tuning, testing) performed on the WESAD dataset, to establish an efficient stress detection algorithm. The task of stress detection was addressed as a binary classification problem.

##### Oversampling

The challenge of data imbalance—i.e., the unequal distribution of classes within the dataset, with stress instances being outnumbered by baseline instances—was addressed. For data augmentation purposes, the Synthetic Minority Oversampling Technique was applied, as implemented by SMOTE class provided by the imbalanced-learn Python library, https://pypi.org/project/imbalanced-learn/, accessed on 20 December 2022.

##### Training and Testing

A multi-subject training dataset, consisting of 12 subjects, was constructed. The remaining 3, previously unseen subjects, were held out for testing purposes. For training purposes, cross-validation was performed, using a stratified shuffle split cross-validator, so that the folds were made by preserving the percentage of samples for each class (stress vs baseline). Prior to proceeding with a suite of machine learning algorithms, a reference accuracy score equal to 64% was established, by using the family of dummy classifiers provided by the scikit-learn library—specifically, the classifier always returning the most frequent label (strategy = ’most frequent’) on the 3-subject test dataset. This baseline accuracy score served as a reference point. Regarding the experimentation set-up, three approaches were followed, for training and testing a pipeline object:

- training/testing a classifier;
- training/testing a 2-step pipeline consisting of a scaler object and a classifier;
- training/testing a 3-step pipeline, consisting of a scaler object, a dimensionality reduction step implemented by Principal Component Analysis (PCA), and a classifier.

Different algorithms exploited for implementing the listed steps are reported in Table 4.

Different hyper-parameter configurations were tested, using GridSearchCV, https://scikit-learn.org/stable/modules/generated/sklearn.model_selection.GridSearchCV.html, accessed on 20 December 2022, i.e., the technique of performing an exhaustive search over a user-specified parameter grid for an estimator. The methodology followed for hyperparameter tuning was to initiate the process with sparse grids of relatively few steps, and to define a finer grid upon identification of a parameter subspace yielding a satisfying performance. The Grid used for the hyper-parameter testing is presented in Table A1 of Appendix A.

##### Selected Metrics for Model Evaluation

A combination of two classification performance metrics, each one serving a different purpose, was selected for properly evaluating the performance of the modeling approach. Classification accuracy, i.e., the function giving the proportion of correctly classified instances, is the most-often reported metric, and as such it is reported here also, for comparability purposes. The F1 score was the second metric to be employed: defined as the harmonic mean between recall and precision, this was the metric preventing the model from maximizing either recall or precision at the expense of the other metric. In the use case under study, the occurrence of a false negative meant that a person in actual need of help during the emergency failed to receive the vital assistance they needed. The occurrence of a false positive caused sub-optimal resource allocation, by placing the focus on persons that could actually cope on their own. Consequently, keeping both false negatives and false positives at a manageable rate of occurrence was considered significant.

#### 3.3.5. Laboratory Study for Operational Testing of the Stress Detection System

##### Aim

To elicit a general physiological stress response based on well-acknowledged stressors, widely used to consistently manipulate stress levels in a laboratory setting. This would serve the purpose of a preliminary validation of the real-time stress inference performed on the smart wearable device.

##### Subjects and Preparation

A total of 15 participants (10 males, 5 females), between 26 and 40 years old, were recruited. The experiment was designed to last approximately twenty minutes for each participant, and was performed in a closed laboratory. Upon their arrival, the participants were kindly requested to wear the smart wearable device. The participants were informed that they would go through a relaxation procedure prior to their undergoing a series of brief tasks, and that they could terminate the experiment at any time they desired. After being outfitted with the equipment, the experiment was initiated, along with the data recording session.

##### Stressor

Stressors employed in pertinent work mainly fall under three categories:

- cognitive category: triggering stress responses via tasks requiring significant mental engagement and focus (e.g., performing an arithmetic task);
- social-evaluative category: triggering stress responses via the ’threat’ of being negatively judged by others (e.g., delivering a public speech in front of a panel while being evaluated by it);
- physical category: triggering stress responses via subjecting the participant to a physically uncomfortable situation (e.g., placing the subject’s hand into a bucket of cold water, and leaving it there for a predefined number of seconds).

The Trier Social Stress Test (TSST), which is the stress elicitation protocol adopted in the context of WESAD data collection, is a well-established laboratory procedure that combines stressors from cognitive and social-evaluative categories [64]. As the machine learning pipeline was developed based on the WESAD dataset, the authors proceeded with a similar, but not identical, combination of stressors. Specifically, the following two tasks were used, to induce cognitive load:

- a temporally constrained arithmetic task, during which the subjects had to count backward from 2485, subtracting 13 for two minutes, and start over upon error occurrence;
- a Stroop Color Word test (SCWT) [65], where a color name was written in a color other than its meaning. The subjects were presented with multiple (four) choices of letters, and were requested to click on the initial letter of the color they actually saw.

SWCT is a widely employed stress-eliciting protocol [66,67,68].

##### Ground Truth Acquisition

For the needs of the current study, each time the participants completed a task, they were kindly requested to respond to a 4-point Likert-scaled question regarding the stress levels they experienced during the task they were previously subjected to (no stress/low stress/moderate stress/high stress).

##### Study Protocol

The experiment was composed of a baseline and a stress phase combined with a self-assessment process. Tasks consisting of the stress phase were separated by a 2 min recovery phase. In the context of the baseline phase, the participants were asked to sit in a relaxed position, and watch a relaxing music video. The stress-inducing part of the study consisted of performing the mental task and a Stroop Color Test, as described in the previous subsection. The timeline of the experiment is presented in Table 5.

##### Integration to Microcontroller

The final, yet very important, step of the whole process was the porting of the model into the smart wristband, and its successful execution by the wristband’s MCU in real time. To successfully host and execute the whole pipeline into the core of the device, the migration of the feature extraction and classification methods into the MCU had to be completed, as the preprocessing phase was already part of the firmware. To achieve this, a process of converting the software for these two parts, written in Python language, to lower-level software in C language, was essential. During the implementation of this task, plenty of challenges arose, due to the fact that the microcontroller unit had restrained resources in regard to memory and computing power. We overcame these challenges with multitasking operations, proper kernel scheduling, and memory management.

Specifically, for the feature extraction part, four functions were developed in C language, based on the spicy Python library: find_peaks(); fir_filter(); kurtosis(); and skew(). The CMSIS DSP library for Arm, https://www.keil.com/pack/doc/CMSIS/DSP/html/index.html, accessed on 22 December 2022, was used for this task.

Regarding the classification part, the Support Vector Machine (SVM) classifier was used, as mentioned in the previous sections. SVM is a popular supervised classification algorithm, which attempts to fit an optimal hyperplane that separates each class from the other [69]. The separating hyperplane is described by

(3) wTxs¯1≷0c

and defines in which half of it every new sensed data vector $x_{s}$ lies. In Equation (3), the coefficients of the hyperplane are expressed by the *d* × 1 floating-point vector *w*, the *c* is the negative of the intercept of the hyperplane, and $\bar{x_{s}}$ is the standard scaled transformed vector $x_{s}$, which is calculated as:

(4) $$\bar{x_{s}}\left[i\right]=p\left[i\right]\left(x_{s}\left[i\right]-u\left[i\right]\right),\forall i\in \left\{1,2,...,d\right\}$$

where *u* and *p* are the d-length floating-point arrays, having the means and the inverse of the standard deviations of the training samples, respectively. The SVM is a scale-variant algorithm, so the scaling of the data was necessary. It must be noted that the linear SVM was preferred to the kernelized non-linear one, because the inference of the latter was too complex for a resource-constraining device. Regarding the time complexity of the linear SVM, 2 *d* multiplications, 2 *d*—1 additions/subtractions, and a single comparison are required for an inference operation.

To implement all the above in real time, micro- learn, https://pypi.org/project/micro-learn/, accessed on 22 December 2022, a Python library for converting machine learning models trained using scikit-learn into inference code, was exploited [70]. This library generated a .c file, with a size of only 1.23 KBytes, that included the necessary arrays, p, u, and w, as described in Equations (3) and (4). Although the conversion to a lower-level code reduced the decimal accuracy of the calculations, due to the reduction floating-point precision of 64-bits in python to 32-bits in C, the performance of the SVM classifier was not affected as outlined in [71]. To evaluate the performance of the integration of the model in the MCU, the processing time required for all signal processing, feature extraction, and classification steps was measured by executing manual tests with a given input (signal of 3000 PPG values) and real-time tests with PPG values acquired from multiple persons’ wrists. The processing execution time measured was in the range of 150–180 ms, which concluded with the real-time stress assessment functionality.

## 4. Results and Discussion

### 4.1. Machine Learning Pipeline Evaluation: Development Phase

The top performers of the training process were Support Vector Machines, Decision Trees, and ensemble algorithms. Specifically, classification with Support Vector Machines, Decision Trees, and ensemble algorithms achieved performances (both in terms of accuracy and F1 score) within a range of 87–91%, with the Extra Trees classifier outperforming the rest of the algorithms. As expected, the non-ensemble category performed significantly better when combined with a scaler step, while the latter exhibited high performance even when used as a standalone component. However, in several cases, combining ensemble algorithms with scalers and a PCA component boosted the performance even more. The post-hyperparameter tuning performance of the candidate pipelines on the held-out 3-subject test dataset (not to be confused with the validation dataset exploited at the training phase for cross-validation purposes) is reported in Table 6.

As shown above, pipelines containing ensemble algorithms outperformed pipelines containing Support Vector Machines and Decision Trees algorithms. Specifically, the top performer of the ensemble family algorithms was XGBoost combined with a min–max scaler and a PCA component, while the top performer of the non-ensemble algorithms was linear Support Vector Machines (combined with robust or standard scaler). The former approach achieved accuracy of 96% versus the 91% attained by the latter. However, given the requirement of real-time stress detection performed at the level of the microcontroller, the authors opted for the non-ensemble approach, i.e., for the pipeline consisting of Support Vector Machines with a linear kernel combined with a standard scaler object.

### 4.2. Machine Learning Pipeline External Validation

**Figure 13:**
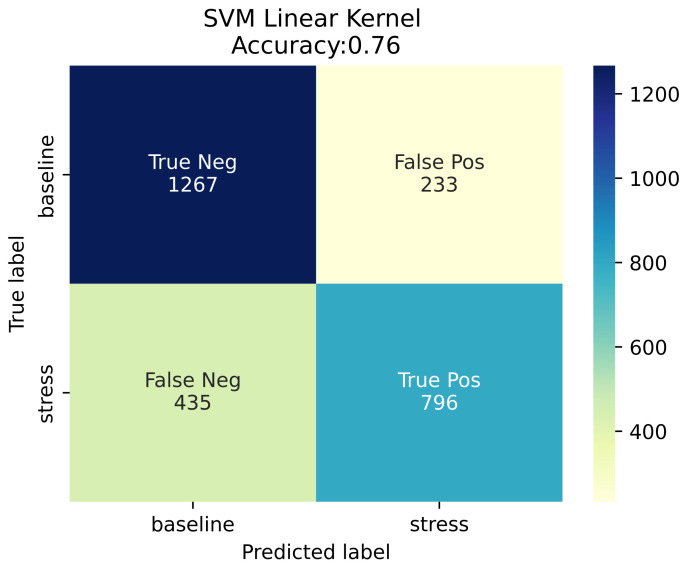
Confusion matrix.

This section reports the detection system performance on the 15-subject dataset collected in a laboratory environment for external validation purposes. At this point, it should be stressed that while the WESAD public dataset provided blood volume pulse data recorded from a commercial wrist-worn device, the annotated signal against which the established stress detection system was validated was a PPG signal recorded with the use of the authors’ wearable device. Additionally, the study protocol adopted for the external validation of the stress system was similar but not identical to the one used by [15] for generating the WESAD dataset. Due to the aforementioned discrepancies, the authors’ realistic goal was not to obtain performance as close as possible to the performance obtained on the held-out test dataset (WESAD), but rather to sustain the expected performance drop within an acceptable range. For each subject and for each task, the system inference was compared to ground truth, as reflected by the corresponding self-report. As described in Section 3.3.5, the participants were kindly requested to respond to a Likert-scaled (1–4) question per task, regarding the extent of the stress they experienced during each task. In order to end up with binary labels a threshold was applied to the extent of the perceived stress level. A ’no stress’ label was assigned to self-assessed stress levels 1 (no stress) and 2 (low stress), while a ’stress’ label was assigned to self-assessed stress levels 3 (moderate stress) and 4 (high stress). The rationale behind the threshold selection was related to the nature of the use case of interest, where ’low’ levels of stress were expected to constitute the baseline. The stress detection system obtained an accuracy as high as 76% and an F1-score equal to 70%, as displayed in the confusion matrix (Figure 13).

## 5. Conclusions

In the context of the presented research work, an AI-enabled smart wristband was designed and developed to serve as a physiological monitoring tool in critical and emergency events, such as the evacuation of a large passenger ship. The implemented wearable device conducts real-time ultra-short PRV analysis and, based on the results, a lightweight machine learning pipeline performs real-time detection of stress. Firstly, research on previous studies focusing on stress detection and the most relevant biosignals was conducted, leading us to the conclusion that the signal generated by PPG technology-based sensors was the most appropriate for our use case. Subsequently, taking into consideration all the concluded system requirements, we proceeded with the acquisition of the appropriate sensor and SoCs that composed the final PCB board of the device. The design and development of the PCB board was a time-consuming yet critically important process that offered reliability, miniaturization, reduced cost, and scalability. Furthermore, the housing of the device was also fully custom-made, and was completed via 3D printing, to fit properly all the hardware sub-components, and to offer convenience to the wearer. Another critical part of the described work was the implementation of the signal preprocessing pipeline and the ML-enabled stress detection model, performing binary classification (“stress” vs “no stress”) of human stress states, based on a unimodal input provided by the PPG sensor. In addition, the integration and real-time execution of the ML model into a resource-constrained component, such as the wristband’s MCU, were achieved. In the end, an evaluation process of the proposed model’s performance was completed in two phases. The first phase of the evaluation was conducted on a subset of the WESAD dataset that had not been used in the training process, achieving 91% accuracy. The second phase of the evaluation was performed on an independent dataset acquired during a dedicated laboratory study, where 15 participants were subjected to specific cognitive stressors while wearing the smart wristband. On this occasion, the smart wristband managed to reach an accuracy score equal to 76%.

Even though the reported 76% was lower than the performance obtained by several of the related approaches presented, it should be stressed that the latter:

- were based on multi-modal datasets using multiple signals to deduce stress inference, or
- had used an ECG signal, which is considered to be of superior quality compared to the PPG signal, or
- had based their analyses upon longer signal segments (>1 min), or
- combined in parallel two or all of the aforementioned bullets.

Last, but not least, as is also stressed in Section 1.1, the performance often reported by the research community is the one obtained in the context of cross-validation and not using a dedicated test dataset, let alone an external validation process. Overall, the authors of the presented work claim that, given the inelastic requirements imposed by the resource-constraining nature of the use case addressed and, specifically, by the necessity for real-time stress inference conducted on the embedded device, a good trade-off between stress detection performance and induced computational complexity was obtained.

At this point, it is important to highlight that the developed wearable device is not a medical device; hence, the generated results should not be used for diagnostic purposes or medical reports. Nevertheless, the developed wearable device is a research-oriented tool that aims to provide basic physiological indicators, and is a first attempt at real-time stress detection. The presented solution has been intensively evaluated for the time being, within the context of the EU-funded project SafePASS. Updated versions of the solution will also continue to be evaluated for research purposes, and for results with increased reliability.

### Limitations and Future Work

It is acknowledged that the proposed solution provides binary inference, and is not a multi-level stress classification, due to the lack of a dataset supporting a sufficiently fine-grained granularity. In addition, the number of subjects was limited, and the measurements included in the WESAD dataset were obviously obtained by a different medical device, and not by the custom-made device introduced by the authors, i.e., by a different PPG technology-based sensor. As it turned out, the size and nature of the dataset used for the training of our model did not only affect the architecture of the model but also its performance on external unseen data acquired from a different device. Furthermore, the need to provide real-time stress state detection—thus executing the model’s inference on a resource-constrained core like the device’s MCU—narrowed down the options for machine learning classification algorithms, and led us to develop an ultra-short and lightweight solution. Hardware-wise, the emergent restrictions were mainly in regard to the size of the device, and the available budget: these restrictions were tackled by selecting relatively low-cost yet reliable components, and by designing a miniaturized PCB board to host them.

The presented work could be extended by creating a multimodal smart wristband equipped with a multilayer stress classification model. The first step would be to add biometric sensors to the PCB of the device, such as EDA and body temperature sensors, capable of providing biosignals and bio-features that have proven useful in the stress detection problem. Another important addition would be the replacement of the existing MCU with a more powerful one, equipped with extended memory, to enable the hosting of more complex and computationally demanding machine learning algorithms. Finally, further experimentation in motion artifacts removal and correction is being considered for future developments. The aforementioned updates could lead to the research and implementation of a stress classification model, trained on data from multiple biosignals, that would output a multilevel result. Within that scope, alternative and more complex signal prepossessing and classification methods, such as frequency domain features and ensemble algorithms, could be incorporated into the model, to achieve higher performance.

## Figures and Tables

**Table 1:** System Architecture components.

Component	Description
Main Processing Unit (MCU)	A low-power microcontroller that is the main processing unit of the device, and supports multiple communication interfaces with the peripherals (BLE and NFC).
PPG-based biometric sensor	A pulse oximeter and a heart rate sensor that uses PPG to detect blood volume changes and to acquire the heart’s biosignal.
Power management unit	Composed of a lithium polymer rechargeable battery and a magnetic reed switch to power on/off the device.
BLE client device	Gateway supporting NFC and BLE protocols for gathering the biometric data transmitted from the wristband.

**Table 2:** Biometric Sensor Configuration.

LEDs	Pulse Amplitude	Pulse Width	Sample Rate	Sample Averaging
IR	20.6 mA	411 μs	400 Hz	4
RED	20.6 mA	411 μs	400 Hz	4
GREEN	40.032 mA	411 μs	400 Hz	4

**Table 3:** PRV-extracted time domain features.

Features	Desciption
mean_IBI	mean value of IBIs
std_IBI	standard deviation of IBIs
no_of_peaks	number of detected peaks
RMSSD	root mean square of successive differences of IBIs
kurtosis_IBI	kurtosis of the IBIs statistical distribution
skewness_IBI	skewness of the IBIs statistical distribution

**Table 4:** Pipeline steps combined.

Scaling	Dimensionality Reduction	Classification
Standard Minmax Robust	Principal Component Analysis	Linear (Linear Discriminant Analysis) Non-linear (K-Nearest Neighbor, Support Vector Machine, Decision Tree, Naïve Bayes) Ensemble (Bagging, Random Forest, Extra Trees, Gradient Boosting, eXtreme Gradient Boosting, Catboost, Light Gradient Boosting)

**Table 5:** Timeline of the stress-inducing experiment.

Phase	Duration	Experiment
Baseline	3 min	Watching relaxing music video
Self-assessment	1 min	Fill in questionnaire
Stress	3 min	Countdown task
Self-assessment	1 min	Fill in questionnaire
Recovery	2 min	Relaxing
Stress	3 min	Stroop Color Test
Self-assessment	1 min	Fill in questionnaire
Recovery	2 min	Relaxing

**Table 6:** Performance metrics per machine learning model/pipeline.

Machine Learning Model/Pipeline	Accuracy	F1 Score
Robust + SVM	0.90	0.87
Standard + SVM	0.90	0.86
Robust + SVM (linear kernel)	0.91	0.88
Standard + SVM (linear kernel)	0.91	0.88
Robust + PCA + DT	0.88	0.83
ET	0.92	0.88
Robust + PCA + LGBM	0.95	0.92
Robust + PCA + BAG	0.93	0.90
Robust + PCA + GB	0.93	0.90
Standard + PCA + GB	0.94	0.91
Minmax + PCA + GB	0.93	0.90
MinMax + PCA + CatBoost	0.93	0.91
Standard + PCA + XGB	0.93	0.91
Minmax + PCA + XGB	0.96	0.94

## Data Availability

Data sharing not applicable.

## Abbreviations

The following abbreviations are used in this manuscript:

PPG	Photoplethysmography
$SpO_{2}$	Oxygen Saturation
ECG	Electrocardiogram
BVP	Blood Volume Pulse
ANS	Autonomic Nervous System
PR	Pulse Rate
IBI	Interbeat Intervals
HRV	Heart Rate Variability
PRV	Pulse Rate Variability
EMG	Electromyogram
EDA	Electrodermal Activity
PVB	Premature Ventricular Beat
GSR	Galvanic Skin Response
ST	Skin Temperature
STL	stereolithography
BLE	Bluetooth Low Energy
MAC	Media Access Control
MCU	Microcontroller Unit
SoC	System on Chip
PCB	Printed Circuit Board
SMT	Surface Mount Technology
TPU	Thermoplastic Polyurethane
SCWT	Stroop Color Word Test
PCA	Principal Component Analysis
TSST	Trier Social Stress Test

## Appendix A

**Table A1:** Grid used for hyper-parameter testing.

Classifiers	Initial Parameter Grid
SVM	kernel = ’poly’, ’rbf’, ’sigmoid’, ’linear’ C = 60, 50, 40, 30, 20,10, 1.0, 0.1, 0.01, 0.001 gamma = ’scale’, ’auto’
DT	max_depth = 5, 10, 20, 30, 35, 40, 45 min_samples_leaf = 1, 2, 3, 4, 5, 10, 20, 50, 100 criterion = ’gini’, ’entropy’
BAG	n_estimators = 5, 50, 100, 500 max_features = 1, 2, 3, 4, 5, 6 max_samples = 150, 200, 250, 300, 350
GB	n_estimators = 5, 50, 100, 500 max_features = 1, 2, 3, 4, 5, 6 max_depth = 1, 20, 40, 60, 80, 100
ET	n_estimators = 5, 50, 100, 500 max_depth = 1, 10, 20, 40, 60, 70 min_samples_leaf = 1, 2, 3, 4, 5, 10, 20, 50, 100 max_features = 1, 2, 3, 4, 5, 6
LGBM	lambda_l1 = 1, 50, 100 lambda_l2 = 1, 50, 100 min_gain_to_split = 0, 1, 3, 5, 10, 12, 15 min_samples_leaf = 1, 2, 3, 4, 5, 10, 20, 50, 100 bagging_fraction = 0.2, 0.4, 0.6, 0.8, 1 feature_fraction = 0.2, 0.4, 0.6, 0.8, 1 max_depth = 1, 10, 20, 40, 60, 70
CatBoost	learning_rate = 0.001, 0.002, 0.005, 0.007, 0.1, 0.3 l2_leaf_reg = 1, 2, 4, 6, 8, 10 random_strength = 2, 4, 6, 8, 10 depth = 2, 4, 6, 8, 10
XGBoost	alpha = 0, 100, 500, 1000 eta = 0.1, 0.3, 0.5, 0.8 num_round = 1, 1000, 2000, 4000 subsample = 0.5, 0.7, 1 min_child_weight = 0, 50, 100, 120 max_depth = 2, 6, 8, 10, 20
